# Anti-Tumor Effect of *Pinus massoniana *Bark Proanthocyanidins on Ovarian Cancer through Induction of Cell Apoptosis and Inhibition of Cell Migration

**DOI:** 10.1371/journal.pone.0142157

**Published:** 2015-11-05

**Authors:** Jia Liu, Jing Bai, Guoqiang Jiang, Xinli Li, Jing Wang, Dachang Wu, Lawrence Owusu, Ershao Zhang, Weiling Li

**Affiliations:** 1 Department of Biotechnology, Dalian Medical University, Dalian, Liaoning, China; 2 Department of Biochemistry and Biotechnology, Kwame Nkrumah University of Science and Technology, Kumasi, Ghana; 3 Department of Pharmaceutical Sciences, University of South Florida, Tampa, United States of America; Institute of Biochemistry and Biotechnology, TAIWAN

## Abstract

*Pinus massoniana* bark proanthocyanidins (PMBPs), an active component isolated from *Pinus massoniana* bark, has been reported to possess a wide range of biochemical properties. Here, we investigated the anti-tumor effect of PMBPs on ovarian cancer. The results indicated that PMBPs significantly reduced the growth of ovarian cancer cells and induced dose-dependent apoptosis. The underlying mechanisms involved were elucidated to include the loss of mitochondrial membrane potential, down-regulation of the anti-apoptotic protein Bcl-2 and the activation of Caspase 3/9, suggesting that PMBPs triggered apoptosis through activation of mitochondria-associated apoptotic pathway. In addition, wound healing and transwell chamber assays revealed that PMBPs could suppress migration and invasion of ovarian cancer cells. PMBPs dramatically inhibited MMP-9 activity and expression, blocked the activity of NFκB and the activation of ERK1/2 and p38 MAPK. Our findings suggest that PMBPs has the potential to be developed as an anti-tumor drug for ovarian cancer treatment and/ or disease management.

## Introduction

Ovarian cancer remains the fifth most common type of cancer in females and the leading cause of death in gynecologic malignancies [1].The first-line treatment of ovarian cancer is usually traditional surgery combined with platinum-paclitaxel based chemotherapy [2]. Despite the good initial response in most ovarian cancer patients, the 5-year survival rate is still low due to relapses- a common occurrence after first line treatment [3]. There have been few developments in ovarian cancer treatment since the standardization of cisplatin and paclitaxel drugs in the last 20 years [4]. There is an urgent need to develop novel effective drugs for ovarian cancer treatment.

Currently, some natural bioactive phytochemical are used to impede proliferation or metastasis of cancer cells [5]. Such phytochemical are non-toxic and devoid of side effects, thus they are good alternatives and/ or complementary to conventional cytotoxic chemotherapy [5]. The pine bark extract was previously considered an inconvenient waste product in the timber industry but now acknowledged as rich in natural proanthocyanidins with potential medicinal properties [6]. It has been demonstrated that proanthocyanidins can be employed to prevent tumor due to their ability to modulate the activity of multiple targets involved in carcinogenesis [7]. Therefore, the anti-tumor activity of pine bark extract has received the most attention recently.

*Pinus massoniana* Lamb of the Pinaceae family is native to the south and southwest of China. Its bark has been used in traditional Chinese medicine for the treatment of inflammation, arthralgia, rheumatism and cancer [8]. The principal component of *Pinus massoniana* bark is also proanthocyanidins. Its anti-tumor activity have been demonstrated in human hepatoma Hep G2 cells, cervical cancer Hela cells and murine sarcoma S180 cells [9,10,11]. However, the effects of *Pinus massoniana* bark proanthocyanidins (PMBPs) on ovarian cancer and the mechanisms underlying the process are yet to be delineated.

In this study, we evaluated whether proanthocyanidins from *Pinus massoniana* bark could exert anti-tumor effects on ovarian cancer cells and further investigated the detailed mechanisms underlying this process. Our data may provide a basis for the future development of PMBPs as an effective drug for ovarian cancer treatment.

## Materials and Methods

### Materials, reagents and chemicals

Antibodies against caspase-3, caspase-9, Bcl-2 were purchased from Cell Signaling Technology (Denver, MA). Antibodies against MMP-9, NFκB, IκBα and β-actin were obtained from Protein Tech Group, Inc. (Chicago, USA). Antibodies against phosphorylated IκBα, ERK1/2, p38 and JNK were obtained from Sangon Biotech, China. The enhanced chemiluminescence (ECL) kit was purchased from Amersham Life Science, Inc. (United States). The Annexin V-conjugated FITC apoptosis detection kit and JC-1 mitochondrial membrane potential detection kit were purchased from NanJing KeyGen Biotech Co., Ltd (Nanjing, China). Transwells were purchased from BD Biosciences (San Jose, USA). MTT (3-(4,5-dimethyl-2-yl)-2,5-diphenyl tetrazolium bromide) and DAPI (2-(4-amidinophenyl)-6-indolecarbamidine dihydrochloride) were obtained from Sigma Chemical Co. (St. Louis, MO). PMBPs powder was purchased from Shaanxi Sciphar Hi-tech Industry Co., Ltd (Shanxi, China). It contained approximately 95% proanthocyanidins, and stable for at least two years at 4°C.

### Cell lines and cell culture

Ovarian cancer cell line A2780 was obtained from American Type Culture Collection (ATCC) and grown in DMEM medium supplemented with 10% FBS (both purchased from Gibco BRL, Grand Island, NY, USA). OV2008 was kindly provided by Prof. Shiying Cui (Dalian Medical University, China) and grown in RPMI-1640 medium (Invitrogen, Burlington, ON, Canada) supplemented with 10% FBS. Normal ovarian epithelial cell line IOSE80 was obtained by Canadian Ovarian Tissue Bank and grown in medium 199 (Invitrogen) and MCDB 105 (Sigma) supplemented with 10% FBS. When the cells were 80% confluent, they were harvested by 0.25% trypsinization. The cells were treated with different concentrations of PMBPs with appropriate corresponding controls.

### Cell viability assay

The effect of PMBPs on the viability of cells were detected by MTT assay. The cells (1×10^4^/well) were seeded into 96-well plate and incubated for 24h. Then 200 μl medium containing 0, 10, 25, 50, 75, 100 and 120 μg/ml PMBPs were added into each well. Each concentration of PMBPs was sextupled. After 24 h and 48 h PMBPs treatment, 20 μl MTT (5 mg/mL in PBS) was added to each well and incubated for 4 h. The MTT solution was removed and the formazan crystals were dissolved in 150 μl DMSO. Absorbance of the solution was measured using a Multiskan Ascent plate reader at 540 nm wavelength.

### DAPI staining assay

Approximately 4×10^4^ cells/well of ovarian cancer cells were plated in 6-well plate and subsequently treated with PMBE at 0, 25, 50 and 100 μg/ml for 24 h. Cells in each wells were stained with DAPI before fixing with 3.7% formaldehyde. The cells were then washed with PBS and analyzed by fluorescence microscopy.

### Cell apoptosis by flow cytometry

After treatment with 0, 10, 25, 35 and 50 μg/ml PMBPs for 24 h, ovarian cancer cells were harvested and washed with PBS three times, then incubated with Annexin V-FITC and PI for 10 min in the dark. The cells were detected by a FACS/Calibur flow cytometer (Becton Dickinson, Franklin Lakes, NJ, USA).

### Assay for mitochondrial membrane potential

The changes of mitochondrial membrane potential of ovarian cancer cells were detected by flow cytometry using JC-1 detection kit. After treatment with 0, 25, 35, 50 μg/ml PMBPs for 24 h, cells were harvested and incubated with JC-1 dye for 15min at 37°C according to the manufacturer’s protocol. The cells were detected by a FACS/Calibur flow cytometer.

### Wound healing assay

A2780 and OV2008 cells were seeded into 6-well plates and grown to create a confluent monolayer. The cell monolayer was scratched in a straight line with a p200 μl pipette tip. The plates were washed with PBS to remove detached cells and then incubated with complete growth medium containing 0, 5, 10 and 25 μg/ml PMBPs solution for 24 h. Cell migration was observed under a phase-contrast microscope at 100× magnification at 0 and 24 h post-induction of injury. Migrated cells into the denuded area in each of six random fields were measured and quantified using a computer-assisted microscope.

### Transwell chamber assay

Cell migration and invasion were quantified by the transwell chamber assay. Ovarian cancer cells were treated with 0, 5, 10 and 25 μg/ml PMBPs for 24 h and harvested. 1×10^5^ cells in serum-free DMEM were added to each upper chamber and DMEM with 10% FBS was added to the lower chamber as a chemoattractant. After 24 h incubation at 37°C, cells remaining on the upper surface of the membrane were removed and the cells that migrated to the underside of the membrane were stained with 0.1% crystal violet for 10 min. The migrated cells on the underside of the membrane were counted under a microscope at 100× magnification. Six random fields of each transwell membrane were counted and averaged.

### Gelatin zymography

The activity of MMP-9 was detected by gelatin zymography. The tumor cells were treated with 0, 5, 10, 25 μg/ml PMBPs for 24 h. Then the cell medium was collected and centrifuged to remove cell debris. Equal volume of clarified medium was electrophoresed through 10% SDS-PAGE gel containing 0.1% (w/v) gelatin at 4°C. The gel was washed with washing buffer (50 mM Tris-HCl, pH 7.5, 100 mM NaCl and 2.5% Triton X-100) and subsequently incubated in activation buffer (50 mM Tris-HCl, pH 7.5, 150 mM NaCl, 10 mM CaCl_2_, 0.02% NaN_3_ and 1 μM ZnCl_2_) at 37°C overnight. Then the gel was stained with 0.25% (w/v) Coomassie brilliant blue in 45% (v/v) methanol and 1% (v/v) acetic acid and destained with 10% isopropanol/10% acetic acid (v/v) solution. MMP-9 bands were observed at 92 kDa.

### Preparation of total cell lysate and nuclear fraction

For total cell lysate, the cells were homogenized in RIPA buffer (Beyotime, Jiangsu,China). Cell lysates were centrifuged at 12,000 rpm for 10 min at 4°C and the supernatant was collected. Nuclear fraction was extracted by a modified method (Yeh, 2012). Briefly, cells were treated with buffer A (10 mM HEPES, 10 mM KC1, 0.1 mM EDTA, 1.5 mM MgCl2, 0.2% NP-40, 1 mM DTT, and 0.5 mM phenylmethylsulfonyl fluoride) and nuclear pellets were collected by centrifugation at 3000 rpm for 30 s at 4°C. The nuclear fraction was then extracted by buffer B (20 mM HEPES, 25% glycerol, 1.5 mM MgCl_2_, 0.1 mM EDTA, 420 mM NaCl, 1 mM DTT, and 0.5 mM phenylmethylsulfonyl fluoride).

### Western blot assay

The total cell lysates or nuclear extracts were separated by 10% SDS-PAGE and then transferred onto a nitrocellulose membrane by semi-dry apparatus for 1 h. The membrane was blocked with 5% non-fat milk for 1 h and then incubated with the specific primary antibody solution overnight at 4°C. After incubation with the appropriate anti-species secondary antibody for 1 h, the protein bands were visualized by ECL kit.

### Statistical analysis

Data was statistically analyzed using Student’s t test and presented as mean ± SD for three independent experiments. SPSS 15.0 software was used for analyses. The value of P<0.05 was considered statistically significant.

## Results

### The effects of PMBPs on the viability of ovarian cancer cells

**Fig 1:**
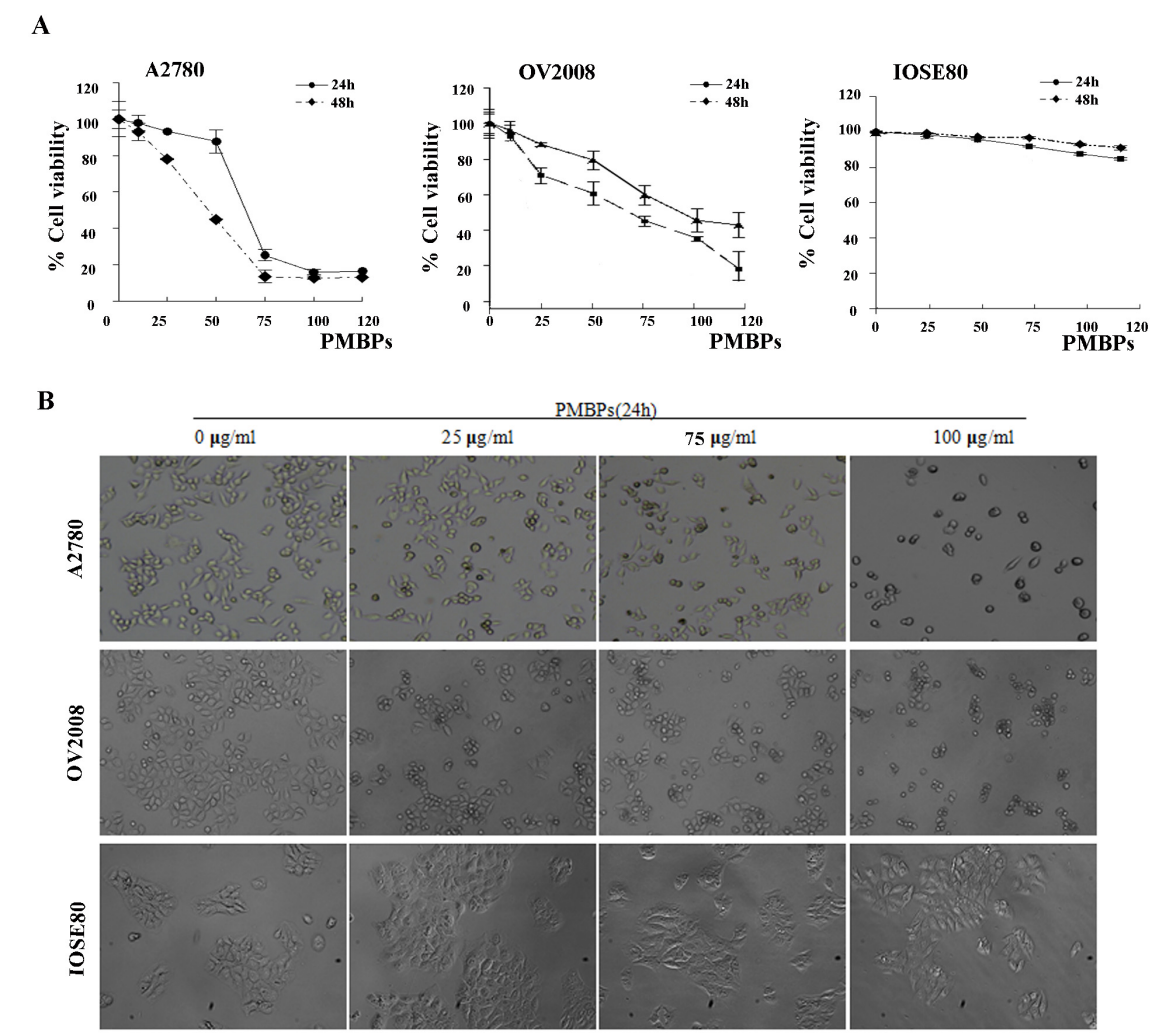
The effects of PMBPs on the viability of ovarian cancer cells and normal ovarian cells. (A) Ovarian cancer cells A2780 and OV2008, and normal ovarian cells IOSE80 were treated with PMBPs (0, 10, 25, 50, 75, 100, 120 μg/ml) for 24 h or 48 h. The cells' viability at the various concentrations of PMBPs were detected by MTT assay and expressed relative to that of non-treated cells. The experiments were repeated three times. (B) Ovarian cancer cells A2780 and OV2008, and normal ovarian cells IOSE80 were treated with PMBPs (0, 25, 75, 100 μg/ml) for 24 h, and cells were photographed with inverted contrast microscopy (magnification, 40×).

The effects of PMBPs on the viability of ovarian cancer cells and normal ovarian cells were firstly detected by MTT assay. Ovarian cancer cells A2780 and OV2008, as well as normal ovarian cells IOSE80 were treated with different concentrations of PMBPs (0, 10, 25, 50, 75, 100, 120 μg/ml) for 24 h or 48 h. Ovarian cancer cells following PMBPs treatment showed a dramatic reduction in cell viability from 25 μg/ml in a dose-dependent manner (*P*<0.01), while same concentrations did not significantly affect the viability of normal ovarian cells IOSE80 (Fig 1A). The IC50 value of PMBPs was 48±2.4 μg/ml for A2780 and 67±2.7 μg/ml for OV2008 at 48 h (Fig 1A).

In addition, the morphologic changes of ovarian cancer cells were examined under a phase contrast microscope. A2780 and OV2008 cells cultured without PMBPs displayed characteristic normal shape with 70% confluence after 24 h. However, the cells' confluency was dramatically reduced with obvious morphologic changes upon PMBPs treatment. The cells started to shrink, lost their normal shape, became round and ultimately detached from the culture dish (Fig 1B). In contrast, we did not observe such morphologic changes in normal ovarian cells, IOSE80, following PMBPs treatment (Fig 1B). All together, these data indicate that PMBPs selectively inhibit the viability of ovarian cancer cells with less effect on nonmalignant cells.

### PMBPs induce ovarian cancer cells’ apoptosis

**Fig 2:**
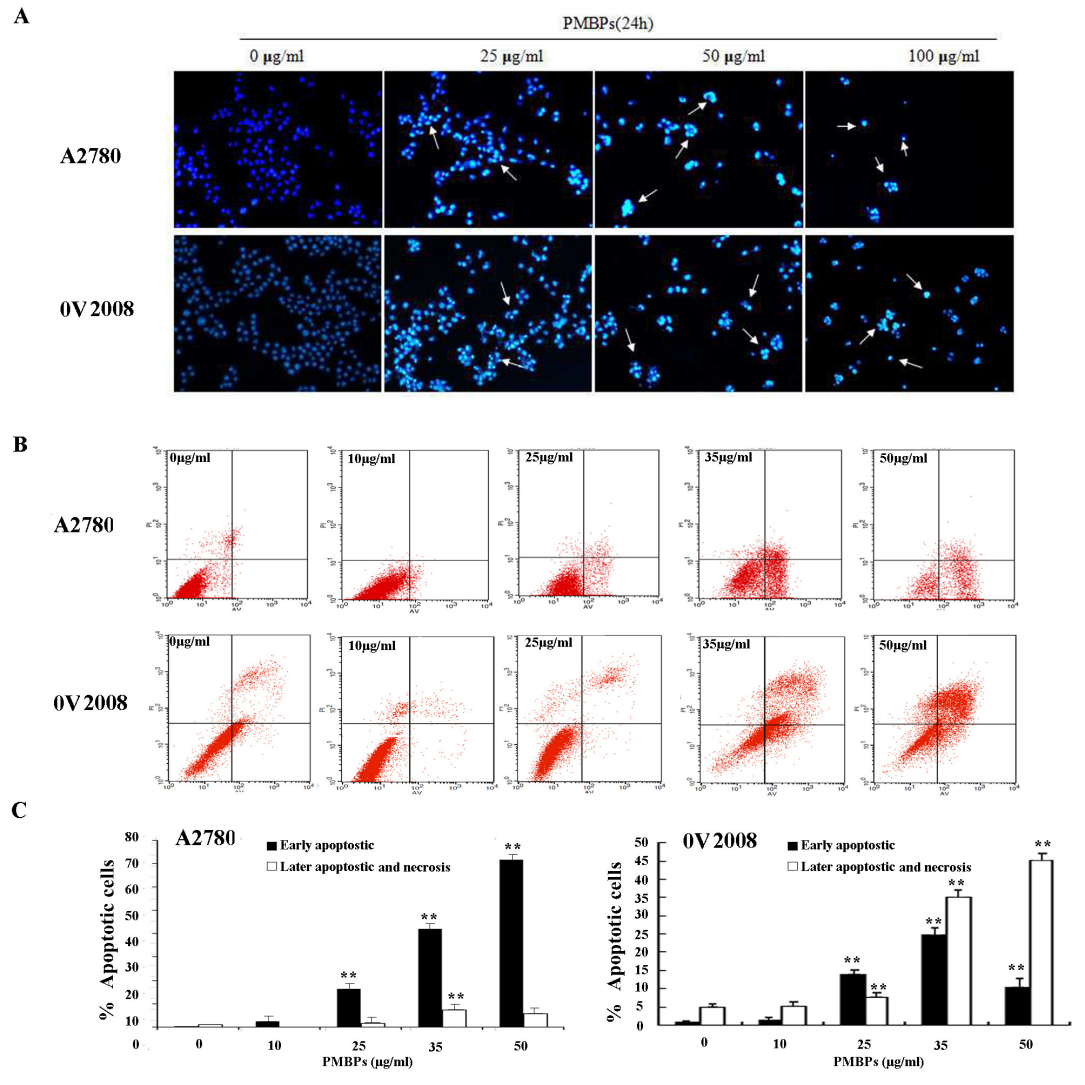
PMBPs induce apoptosis of ovarian cancer cells. (A) A2780 and OV2008 cells were treated with PMBPs (0, 25, 50, 100 μg/ml) for 24 h. The morphological changes of nuclei were examined by fluorescence microscopy using DAPI staining. The arrows indicate nuclear condensation and apoptotic bodies (magnification, 100×). (B) A2780 and OV2008 cells were treated with PMBPs (0, 10, 25, 35, 50 μg/ml) for 24 h. Flow cytometry was applied to analyze Annexin V-FITC/PI double stained A2780 cells. The low right (LR) quadrant of the histograms indicates the early apoptotic cells, and the upper right (UR) quadrant indicates the late apoptotic and necrotic cells. (C) The treatment of A2780 and OV2008 cells with PMBPs resulted in dramatic increase in the percentage of apoptotic and necrotic cells (including early apoptosis, late apoptosis and necrosis). The experiments were repeated three times. **P<0.*01* compared to the control group.

To assess whether the anti-tumor effects of PMBPs on A2780 and OV2008 cells were associated with apoptosis, the ovarian cancer cells were stained with DAPI and observed under a fluorescence microscope (Fig 2A). After treatment with different concentrations of PMBPs for 24 h, nuclear chromatin condensation and fragmented punctuate blue nuclear fluorescence were observed in ovarian cancer cells in a dose-dependent manner, similar to the morphological changes in the apoptotic cells, but the control cells displayed normal and intact nuclei. It suggests that PMBPs may induce apoptosis of ovarian cancer cells.

To further quantify the apoptosis caused by PMBPs, A2780 and OV2008 cells were labeled with Annexin V-FITC/ PI and analyzed by flow cytometry. The lower right quadrant (LR) indicates the percentage of early apoptotic cells (Annexin V^+^ and PI^**-**^) and the upper right quadrant (UR), the percentage of late apoptotic and necrotic cells (Annexin V^+^ and PI^+^). The results showed that PMBPs triggered apoptosis of ovarian cancer cells, starting at 25 μg/ml, in a dose-dependent manner (Fig 2B). The percentage of total apoptotic and necrotic cells of A2780 cells was 1.17% in control cells (LR: 0.2% and UR: 0.97%), 2.46% in cells treated with 10 μg/ml PMBPs (LR:2.42% and UR:0.04%), 17.63% in cells treated with 25 μg/ml PMBPs (LR:16.12% and UR:1.51%), 49.24% in cells treated with 35 μg/ml PMBPs (LR:41.87% and UR:7.37%) and 77.23% in cells treated with 50 μg/ml PMBPs (LR:71.44% and UR:5.79%) (Fig 2B). Similarly, those in OV2008 cells were 5.78% in control cells (LR:0.83% and UR:4.95%), 6.46% in cells treated with 10 μg/ml PMBPs (LR:1.38% and UR:5.08%), 21.45% in cells treated with 25 μg/ml PMBPs (LR:13.88% and UR:7.57%), 59.94% in cells treated with 35 μg/ml PMBPs (LR:24.79% and UR:35.15%) and 55.31% in cells treated with 50 μg/ml PMBPs (LR:10.21% and UR:45.1%)(Fig 2B). Our results demonstrate that PMBPs could significantly induce both early and late apoptosis in ovarian cancer cells (*p*<0.01) (Fig 2C).

### PMBPs induce mitochondrial membrane potential loss

**Fig 3:**
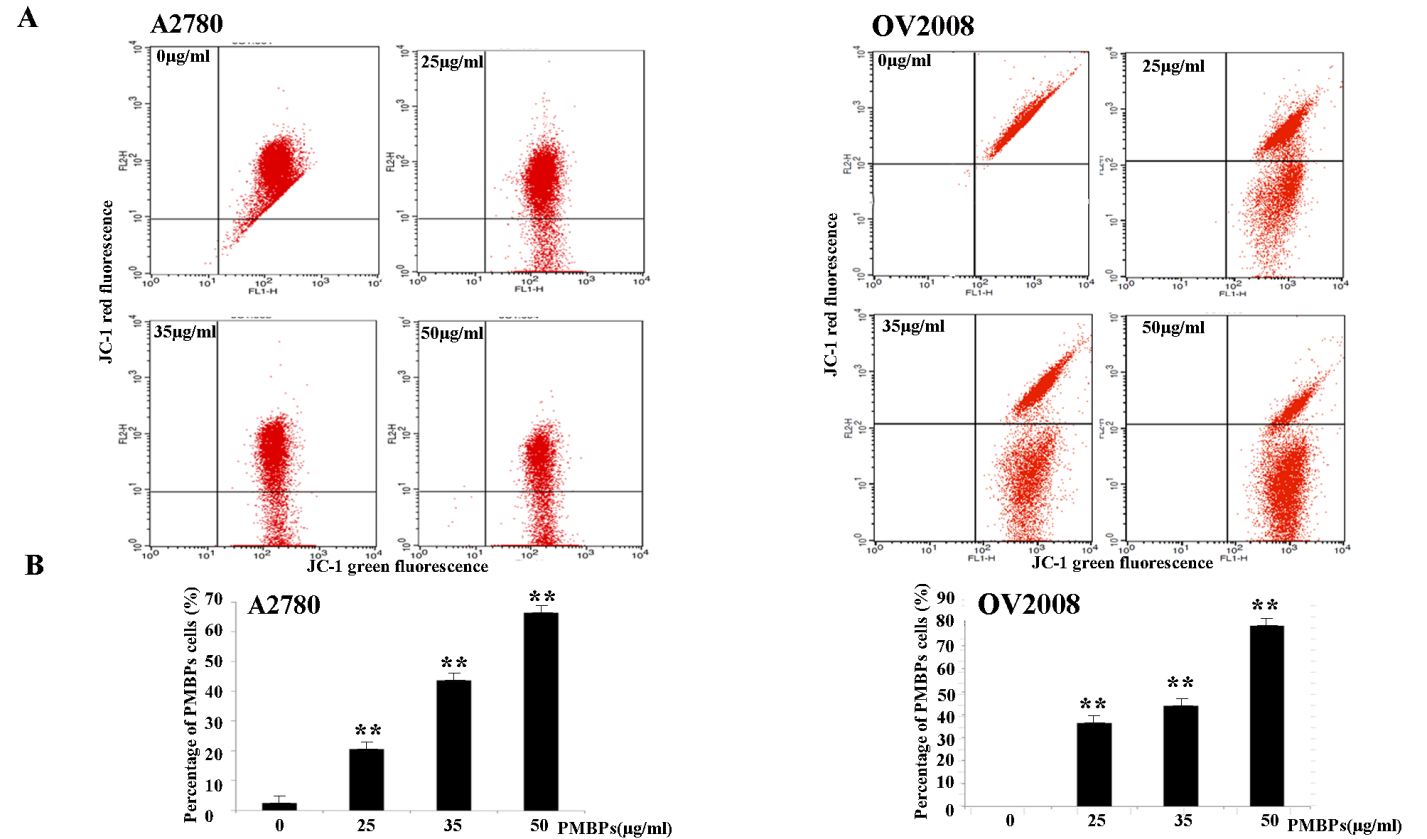
PMBPs induce loss of mitochondrial membrane potential. (A) The A2780 and OV2008 cells were treated with PMBPs (0, 25, 35, 50 μg/ml) for 24 h and then harvested, stained with JC-1 dye, and finally analyzed by flow cytometry. (B) PMBPs treatment resulted in a significant increase of green fluorescence positive (GFP) cells which indicated the loss of mitochondrial membrane potential. The experiments were repeated three times. **P<0.*01* compared to the control group.

It is known that mitochondria damage during apoptosis changes the mitochondrial membrane potential [12]. To explore the effects of PMBPs on mitochondrial membrane potential, the cells treated with PMBPs were detected by JC-1 dye. As shown in Fig 3, the average percentage of green fluorescence-positive A2780 cells was 2.41% without treatment, 20.63% at 25 μg/ml, 43.77% at 35 μg/ml and 66.6% at 50 μg/ml PMBPs treatment. Similarly, those in OV2008 cells were 0% without treatment, 36.28% at 25 μg/ml, 43.81% at 35 μg/ml and 78.79% at 50 μg/ml(Fig 3A). There was significant dose-dependent increase in green fluorescence-positive cells (Fig 3B) (*p<0*.*01*), suggesting the loss of mitochondrial membrane potential correlated with increasing dose of PMBPs treatment. Therefore, the apoptosis of ovarian cancer cells by PMBPs is associated with the damage of the mitochondrial membrane.

### PMBPs upregulate apoptosis-related proteins and suppress Bcl-2

**Fig 4:**
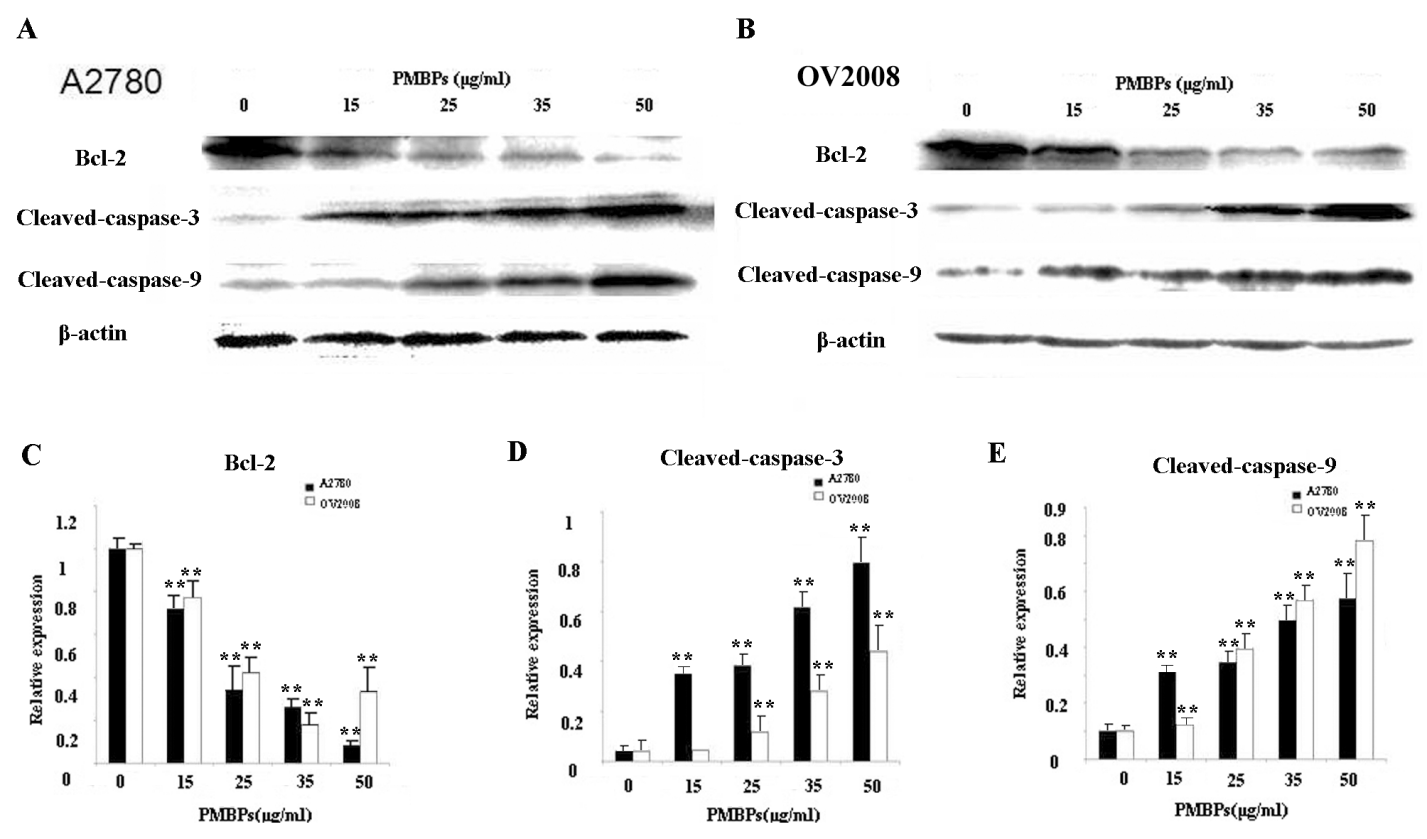
The effects of PMBPs on apoptosis-related proteins. (A,B) The A2780 and OV2008 cells were treated with PMBPs (0, 15, 25, 35, 50 μg/ml) for 24 h and then harvested. Cell lysates were prepared and subjected to Western blot analysis. The protein levels of Bcl-2, cleaved-caspase-3 and cleaved-caspase-9 were measured by Western blot. (C,D,E) Histograms show mean expression levels of Bcl-2, cleaved-caspase-3 and cleaved-caspase-9 (±SD), respectively, from three independent experiments. Bcl-2, cleaved-caspase-3 and cleaved-caspase-9 levels were expressed relative to loading control, β-Actin. All experiments were repeated three times.**P<0.*01* compared to the control group.

Since PMBPs could induce apoptosis in ovarian cancer cells, we further examined some apoptosis related proteins by Western blot. The level of β-Actin served as internal control. We found that the expression of anti-apoptotic protein Bcl-2 in ovarian cancer cells treated with PMBPs significantly decreased in a dose-dependent manner (*p<0*.*01*) (Fig 4A, 4B and 4C). The caspase members are crucial mediators of apoptosis [13]. The expressions of caspase-3 and caspase-9 were assessed. Cleaved-caspase-3 and cleaved-caspase-9 expression levels were dramatically upregulated following PMBPs treatment in ovarian cancer cells (*p<0*.*01*) (Fig 4A, 4B, 4D and 4E). These results suggest that PMBPs activate the caspase-dependent apoptosis pathway.

### PMBPs inhibit the migration and invasion of ovarian cancer cells

**Fig 5:**
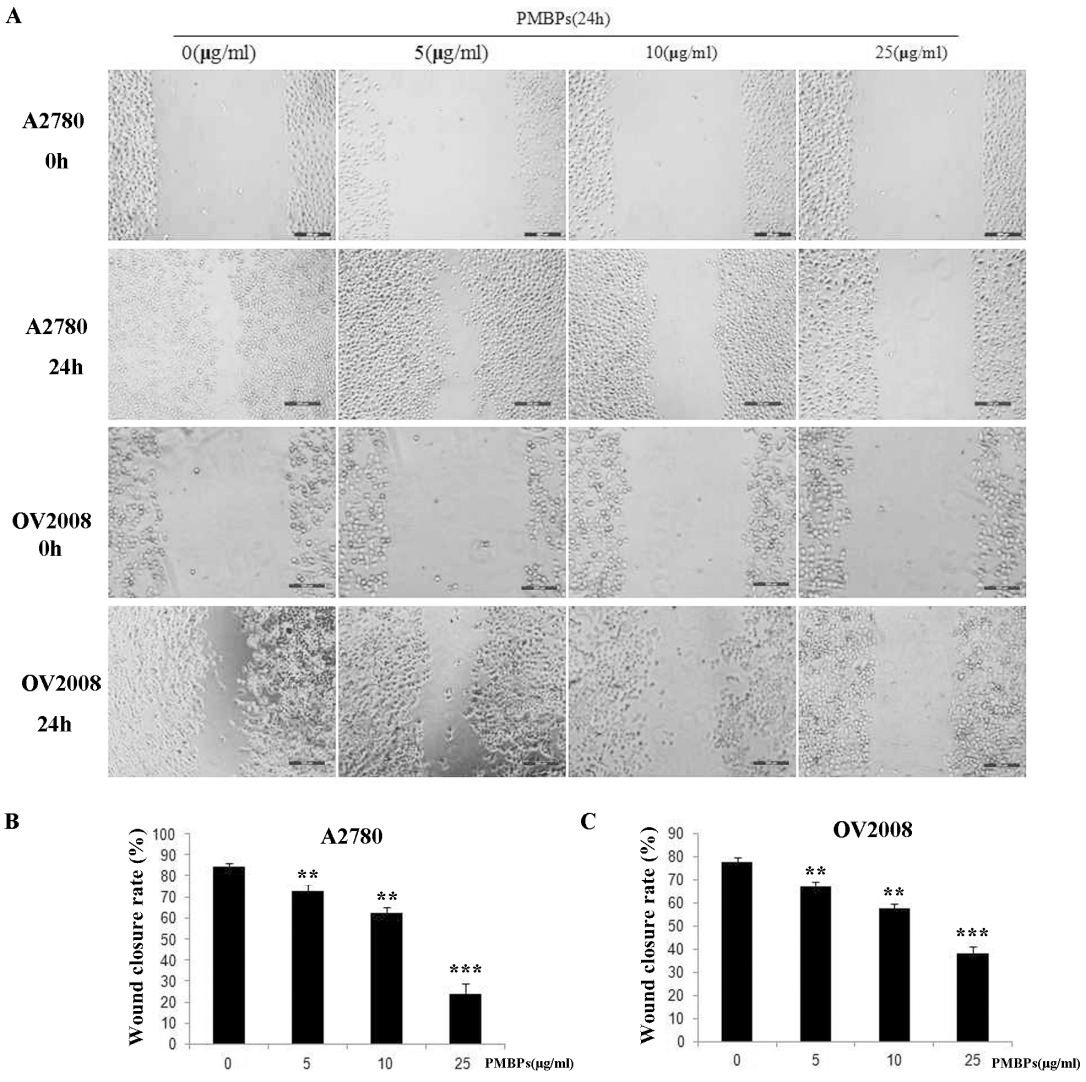
The effects of PMBPs on the migration of ovarian cancer cells by wound healing assay. Monolayers of A2780 and OV2008 cells were scratched with a pipette tip and treated with PMBPs (0, 5, 10, 25μg/ml) for 24 h. (A) Representative photos of migrating cells under microscope at 100× magnification field, before and after injury. (B,C) The migration of A2780 and OV2008 cells was quantified by measuring wound closure areas before and after injury. The experiments were repeated three times. **P<0.*01* and ***P<0.*001* compared to the control group.

**Fig 6:**
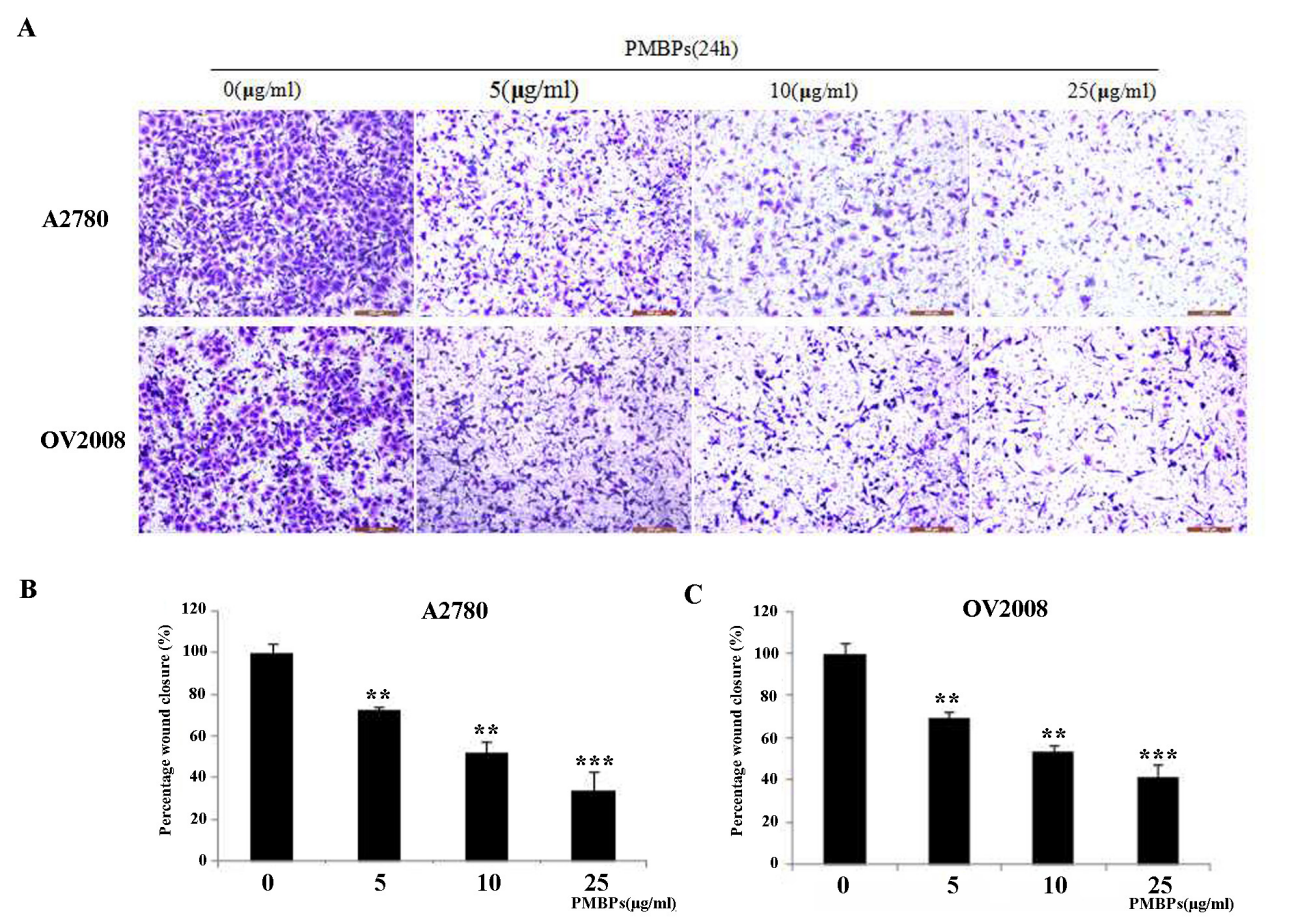
PMBPs impaired the migration and invasion of ovarian cancer cells as assessed by transwell chamber assay. A2780 and OV2008 cells were treated with PMBPs (0, 5, 10, 25 μg/ml) for 24 h. (A) Representative images of cells that migrated and invaded onto the underside of transwell membrane at 100× magnification under a microscope. (B,C) Number of cells of each field was counted and averaged. Invaded cells were expressed relative to that of control group. The experiments were repeated three times. **P<0.*01* and ***P<0.*001* compared to the control group.

Cell migration and invasion are hallmarks of tumor cells. To investigate whether PMBPs impaired migration and invasion of ovarian cancer cells, we performed wound healing assay with different, not apoptotic (5 μg/ml and 10 μg/ml), concentrations of PMBPs for 24 h. We found that PMBPs dose-dependently decreased the movement of A2780 and OV2008 cells (Fig 5). The wound closure rates of PMBPs treated cells were lower than that of non-treated cells (*P<0*.*01*) (Fig 5B and 5C). Additionally, we examined the invasiveness of these cells using a 24-well transwell chamber in the presence of different concentrations of PMBPs for 24 h. PMBPs significantly inhibited the migration and invasion potential of the ovarian cancer cells in a dose-dependent manner (*P<0*.*01*) (Fig 6). Treatment with 5–25 μg/ml PMBPs inhibited migration and invasion by 27%-66%, respectively, in A2780 cells, and 11%-40% in OV2008 cells, compared to controls (Fig 6B and 6C). Both wound healing assay and transwell chamber assay suggest that PMBPs could suppress the migration and invasion of ovarian cancer cells.

### Inhibition of MMP-9 activity and expression by PMBPs

**Fig 7:**
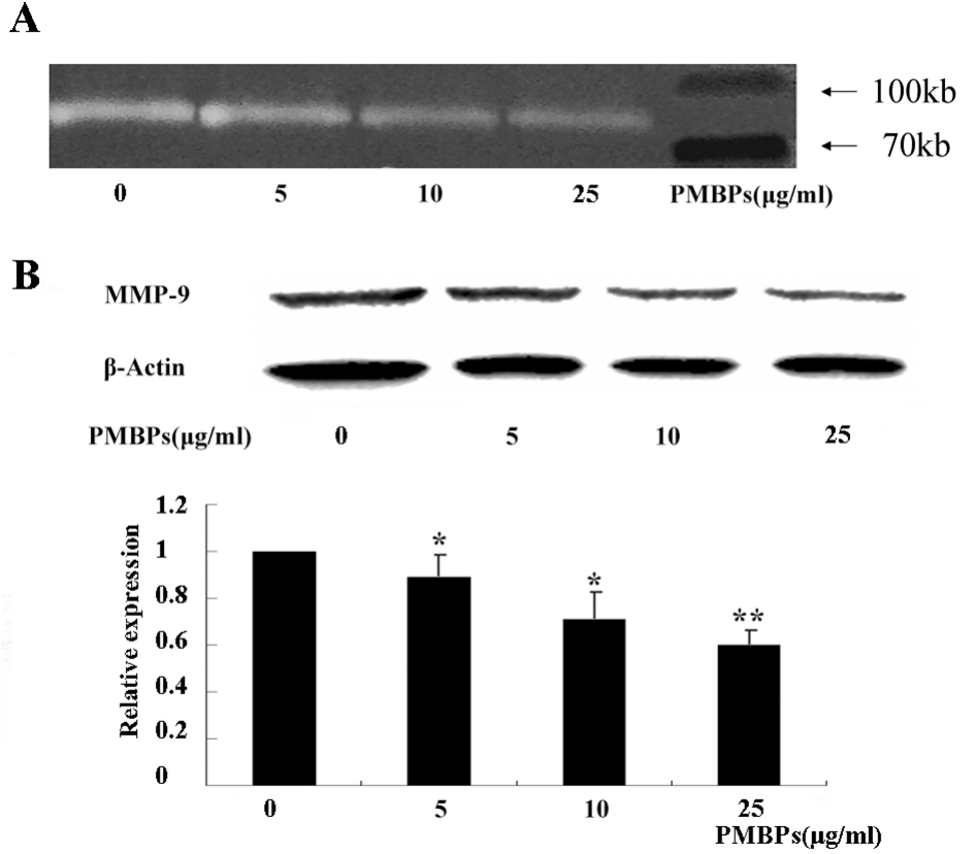
PMBPs suppress MMP-9 activity and expression. The cells were treated with PMBPs (0, 5, 10, 25 μg/ml) for 24 h. (A) Activity of MMP-9 in the cell supernatant at various concentrations of PMBPs was examined by gelatin zymography assay. The white bands represent MMP-9 mediated gelatin digestion. (B) The protein expression of MMP-9 in A2780 cells at various concentrations of PMBPs was evaluated by Western blot. Histogram shows mean level of MMP-9 (±SD) from three independent experiments. MMP-9 protein level was expressed relative to loading control, β-Actin, and standardized to the non-treated control group. *P<0.*05* and **P<0.*01* compared to the control group.

Given the effects of PMBPs on ovarian cancer cell migration and invasion, we further investigated the mechanisms of this process. Since MMP-9 plays an important role in cancer invasion, we next detected MMP-9 enzyme activity and expression after PMBPs treatment. MMP-9 activity in conditioned medium was examined by gelatin zymography, and MMP-9 expression was examined by Western blot. PMBPs dose-dependently suppressed MMP-9 activity (Fig 7A) and reduced MMP-9 protein expression level (*P<0*.*05*) (Fig 7B). Thus, the inhibitory activity of PMBPs on the invasiveness of ovarian cancer might be, at least partially, due to the suppression of MMP-9 activity.

### PMBPs impair NFκB nuclear translocation and MAPK pathway activation

**Fig 8:**
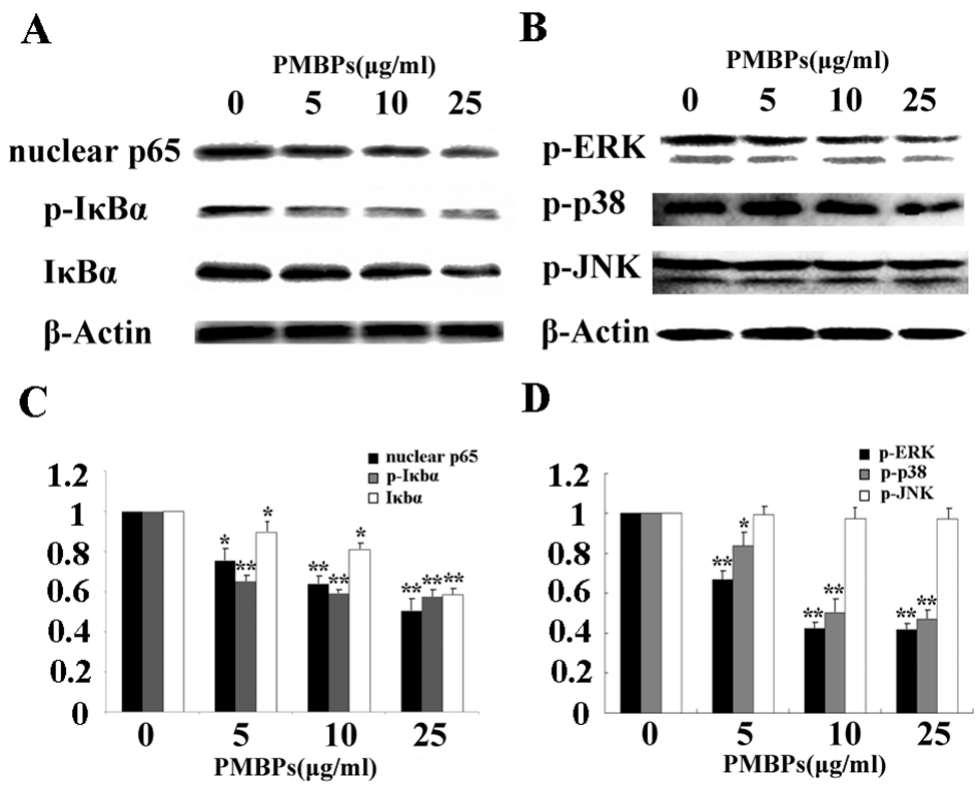
Effect of PMBPs on NFκB activity and MAPK pathways. (A,B) The cells were treated with PMBPs (0, 5, 10, 25 μg/ml) for 24 h. Cell lysates were prepared and subjected to Western blot analysis. The protein level of nuclear p65, p-IκBα, IκBα, p-ERK, p-p38 and p-JNK were measured. (C,D) Histograms show mean (±SD) level of nuclear p65, p-IκBα, IκBα, p-ERK, p-p38 and p-JNK from three independent experiments. The nuclear p65, p-IκBα, IκBα, p-ERK, p-p38 and p-JNK expression levels were expressed relative to loading control β-Actin and standardized to the non-treated control group. *P<0.*05* and **P<0.*01* compared to the control group.

Many studies have demonstrated that inhibiting NFκB activity could suppress cancer cell invasion [14,15,16]. We, therefore, investigated the effect of PMBPs on NFκB activity in the ovarian cancer cells. Treatment of A2780 cells with PMBPs significantly reduced the nuclear translocation of NFκB and phosphorylation of IκBα (*P<0*.*05*) (Fig 8A and 8C). These prompted that PMBPs may, at least in part, suppress ovarian cancer cell invasion by inhibiting NFκB activity. Several studies have indicated that activation of mitgen-activatied protein kinases (MAPKs) including ERK1/2, p38MAPK, JNK are involved in tumor migration and invasion, and that these MAPKs act upstream of NFκB [17,18,19]. Therefore, the activation status of ERK1/2, p38MAPK and JNK was examined in A2780 cells treated with various concentrations of PMBPs. PMBPs significantly inhibited ERK1/2 and p38MAPK activation (*P<0*.*05*) but not JNK (*P>0*.*05*) (Fig 8B and 8D). Thus, the suppression of ERK1/2 and p38MAPK activation by PMBPs may contribute to the impaired invasion potential of the ovarian cancer cells.

## Discussion

Ovarian cancer remains the leading cause of death in gynecologic cancers largely because of late stage diagnosis and limited effective treatments, especially in the recurrent disease [20]. Natural products derived from plants are promising therapeutic agents for cancer treatments [21]. Therefore, we explored the potential utility of proanthocyanidins from *Pinus massoniana* bark on ovarian cancer cells in the quest of finding novel effective drugs for ovarian cancer treatment. Our data here demonstrated that PMBPs reduced the proliferation, induced apoptosis and suppressed the migration of ovarian cancer cells.

*Pinus massoniana* bark extract has been reported to inhibit growth of several kinds of cancer. It selectively prevented the proliferation of human hepatoma BEL-7402 and HepG2 cells but slightly promoted the growth of human normal liver cells L-02 in vitro [8,9,22]. It also suppressed the growth of human cervical cancer Hela cells and induced apoptosis. In this study, we found that proanthocyanidins extracted from *Pinus massoniana Lamb* bark significantly inhibited the proliferation of ovarian cancer cells but did not affect normal ovarian cells. We also observed that cancer cells shrank and detached from culture dishes after PMBPs treatment, which suggests that PMBPs selectively prevent the growth of ovarian cancer cells and cause cell death.

We further investigated the anti-tumor mechanisms employed by PMBPs on ovarian cancer cells. Apoptosis plays a very important role in eliminating mutated or hyper-growing tumor cells [23]. There are several kinds of natural products that have been reported to prevent the growth of tumor cells via apoptosis induction [24]. Some studies have revealed that *Pinus massoniana* bark extract induce apoptosis in human cervical cancer Hela cells, human hepatoma HepG2 cells and murine sarcoma S180 cells in a dose-dependent manner [9,10,11]. In the present study, DAPI staining data revealed that cells treated with PMBPs displayed specific apoptotic morphological changes. Flow cytometry data further indicated that the proportion of apoptotic cells significantly increased following PMBPs treatment. All these results indicate that PMBPs induces apoptosis in ovarian cancer cells.

The induction of apoptosis is related to the suppression of anti-apoptotic proteins and up-regulation of pro-apoptotic proteins [25]. It has been reported that Bcl-2 protein is paramount in regulating the mitochondria-associated apoptotic pathway [26]. Down-regulation of Bcl-2 results in the loss of mitochondrial membrane potential and release of cytochrome *c* from mitochondria to the cytosol to activate caspase-9 [27]. The cleaved-caspase-9 further activates caspase-3, one of the key enzymes in the intrinsic apoptotic pathway, to induce subsequent apoptotic events [28]. Herein, our results showed that PMBPs induced a significant loss of mitochondrial membrane potential. In addition, we observed the reduction of Bcl-2 expression accompanied with elevated levels of cleaved-caspase-9 and cleaved-caspase-3 in ovarian cancer cells treated with PMBPs. It could be considered that PMBPs may induce apoptosis through the mitochondria-associated apoptotic pathway.

We found that PMBPs effectively suppressed ovarian cancer migration and invasion. One important factor that facilitates the metastatic spread of cancer cells is proteolytic enzymes which degrade the surrounding extracellular matrix, and thus facilitate cell migration through the basement membrane. Matrix metalloproteinases (MMPs) are involved in matrix remodeling [29]. With regard to ovarian cancer, the expression of MMP-9 has been linked with invasive subtypes [30]. Our work demonstrates that PMBPs could down-regulate MMP-9 expression and activity. Therefore, PMBPs may suppress MMP-9 activity to impair the migration and invasion capabilities of ovarian cancer cells.

Activation of NFκB is crucial for the migration and invasion of ovarian cancer cells [31,32,33]. We proposed that PMBPs may suppress NFκB activity and inhibit ovarian cancer invasion. The phosphorylation of IκBα can release NFκB subunits which are transported from the cytoplasm to the nucleus of cells to regulate target genes [34]. In this study, we observed that IκBα phosphorylation and NFκB translocation from the cytoplasm to the nucleus inhibited by PMBPs treatment (Fig 8A and 8C). NFκB serves as transcription factor for the MMP-9 regulation [35,36]. Our results demonstrate that PMBPs suppress NFκB activity and this positively correlate with the down-regulation of MMP-9 expression.

The MAPK signaling cascade, including ERK1/2, p38 MAPK and JNK, has been implicated in the migration and invasion of numerous cancer cell types [37,38,39]. In this study, we speculated that PMBPs might antagonize the MAPK signaling pathways to suppress migration and invasion of ovarian cancer cells. Our data indicated that PMBPs treatment inhibited the activation of ERK1/2 and p38 MAPK in a dose-dependent manner, but had little effect on JNK pathway (Fig 8B and 8D). Our data here, together with others, show that MMP-9 can be activated through multiple signaling pathways including the MAPKs family [40,41,42], however, whether PMBPs directly regulated MMP-9 activity through ERK1/2 and p38 MAPK pathways needs further investigation.

In conclusion, PMBPs exert anti-tumor effects on ovarian cancer cells through inhibition of cell growth, induction of apoptosis and suppression of cell migration and invasion in vitro. As the pioneering data to demonstrate PMBPs' efficacy to trigger apoptosis by activating the mitochondria-associated apoptotic pathway and to inhibit cell migration and invasion via MMP-9 down-regulation, PMBPs could be developed as a novel effective anti-tumor agent for ovarian cancer treatment.
